# Burden of osteoarthritis in older adults (aged ≥55 years) in the United States and China: a comparative analysis of temporal trends, risk factor contributions, and projected burden to 2030 using global burden of disease study 2021 data

**DOI:** 10.3389/fmed.2025.1636976

**Published:** 2025-09-16

**Authors:** Xuequan Han, Chi Zhang, Bin Guan, Hengxing Zhou, Xiaohong Kong, Shiqing Feng

**Affiliations:** ^1^Department of Orthopedics, Qilu Hospital of Shandong University, Shandong University Centre for Orthopedics, Advanced Medical Research Institute, Cheeloo College of Medicine, Shandong University, Jinan, China; ^2^Department of Orthopaedics, The Second Hospital, Cheeloo College of Medicine, Shandong University, Jinan, China

**Keywords:** global burden of disease study, osteoarthritis, aging, body mass index, China, United States

## Abstract

**Objective:**

Osteoarthritis (OA) is a leading cause of disability worldwide, with aging populations and rising obesity rates being notable contributing factors. This study examines OA burden trends from 1990 to 2021 among individuals aged 55 and older in China and the United States (US), with projections through 2030, while addressing the gap in comprehensive, site-specific, and age-stratified OA data in the US.

**Methods:**

Data from the Global Burden of Disease (GBD) 2021 study were used to evaluate age-standardized rates of incidence (ASIR), prevalence (ASPR), and disability-adjusted life years (AS-DALYs), as well as absolute numbers and age-specific rates, for OA and site-specific OA. Temporal trends were analyzed using Joinpoint regression, while age-period-cohort (APC) analysis was employed to assess contributing factors. Bayesian APC modeling was applied to project OA burden trends through 2030.

**Results:**

From 1990 to 2021, the OA burden exhibited distinct yet noteworthy trends in the US and China. The US consistently maintained markedly higher age-standardized incidence, prevalence, and disability rates than China, reflecting a substantial and persistent OA burden among older adults. However, China experienced a more rapid escalation in disease burden, especially for hand and hip OA. For example, the average annual percentage change (AAPC) of ASIR and ASPR in China reached 0.46%, surpassing the US (0.20 and 0.16%, respectively). Notably, hand OA prevalence in China showed an exceptionally steep rise (AAPC = 1.6%), far outpacing the US (0.2%). In absolute numbers, China bore a much larger burden due to its population size. Projections to 2030 indicate a continued global increase in OA burden, with the US expected to retain high prevalence and China projected to undergo steeper growth, highlighting differing but significant public health challenges in both countries.

**Conclusion:**

This study demonstrates a persistently high OA burden in the US, while China shows rapidly increasing rates, especially for hand and hip OA in older adults. With differing trajectories projected through 2030, region-specific strategies are warranted: China should focus on curbing accelerating incidence and addressing modifiable risks like obesity, while the US should enhance management to reduce disability in a population already facing high OA prevalence.

## 1 Introduction

Osteoarthritis (OA), a highly prevalent age-related joint disorder, currently affects over half a billion people worldwide (approximately 7% of the population) (1, 2). This number is projected to rise to 15–20% by 2050, primarily due to aging and rising obesity rates (3, 4). OA causes cartilage breakdown within joints, often leading to progressive physical limitations that compromise independence and well-being (5). Age-related joint degeneration heightens susceptibility to cartilage deterioration. Concurrently, obesity imposes mechanical stress on load-bearing joints such as the knees and hips, hastening OA progression (6, 7).

As two of the most influential nations globally, China and the United States (US) face significant OA challenges (8, 9). They have large populations but differ greatly in demographic profiles, cultural practices, lifestyles, and healthcare systems, all of which may influence the incidence and progression of OA. With a population exceeding 1.4 billion, China is undergoing rapid demographic aging—a major risk factor for OA. At the same time, rising obesity rates linked to shifting lifestyles could contribute to an unusually high OA prevalence (10). Conversely, the US has a more gradual aging process but a higher current prevalence of obesity (11). This creates a different set of challenges. Understanding how OA prevalence and patterns vary across these two populations can highlight major contributing factors, enabling targeted disease management strategies. Such insights may also benefit other nations facing similar challenges (12).

The Global Burden of Disease (GBD) Study is a leading epidemiological resource for global disease trends and risk factors. This database delivers essential epidemiological insights, shaping national health planning and healthcare investments globally. While previous studies utilizing GBD data have extensively investigated the OA burden in China, there remains a noticeable gap in the literature regarding the US—particularly concerning site-specific OA burden in older adults aged over 55 years (8, 13–15). Existing GBD-based research on the US has either focused on overall arthritis prevalence or specific subpopulations, without providing a comprehensive analysis of site-level OA metrics in the aging population (16). Furthermore, the current literature lacks comparative investigations between China and the US using consistent methodologies based on the GBD 2021 data. Such cross-national comparisons are critical for understanding how demographic transitions, lifestyle risk factors, and healthcare systems shape OA patterns differently across global regions.

To address this gap, our study provides a systematic comparison of the OA burden in China and the US from 1990 to 2021 using the GBD 2021 dataset. Focusing on individuals aged 55 years and older, we conduct a site-specific analysis across major joint types (hip, knee, hand, and others) and examine trends in age-standardized incidence, prevalence, and disability-adjusted life years (DALYs). We further assess the contribution of high body mass index (BMI) as a modifiable risk factor for hip and knee OA and employ Bayesian age-period-cohort modeling to project future burden through 2030. By integrating temporal trends, demographic stratification, and comparative analysis, our study fills an important gap in the literature and contributes new, actionable insights on the growing OA burden in aging populations.

To guide our investigation into the burden of OA among older adults, we adopted the World Health Organization’s Healthy Aging Framework, which emphasizes functional ability as the central goal of aging-related health interventions (17). This framework highlights the need to not only extend life expectancy but to ensure individuals maintain the ability to meet basic needs, learn and make decisions, be mobile, build and maintain relationships, and contribute to society (18). As a chronic condition influenced by aging, obesity, physical activity, and access to care, OA is particularly suitable for analysis under this model. Our comparative analysis of OA trends in China and the US is thus grounded in this framework, as it underscores the importance of understanding disease burden to support function-focused, equitable public health planning.

## 2 Methods

### 2.1 Overview and source of data

This study employed the Global Burden of Diseases (GBD) 2021 dataset, developed by the Institute for Health Metrics and Evaluation (IHME) at the University of Washington. The GBD study systematically evaluates 371 diseases and 88 risk factors across 204 countries and territories (1990–2021) using standardized age- and sex-adjusted methodologies. Data were obtained from the publicly accessible Global Health Data Exchange (GHDx).^1^ From GBD 2021 dataset, we extracted age-standardized rates (ASRs) and absolute counts of incidence, prevalence, and disability-adjusted life years (DALYs) for OA, including site-specific subtypes (hip, knee, hand, and other OA), from 1990 to 2021. Additionally, we obtained age-specific population estimates (1990–2021) and projections (2022–2030) for China, the US, and globally, as well as the percentage of OA-related DALYs attributable to BMI (1990–2021) for overall OA, hip OA, and knee OA in these regions.

### 2.2 Case definitions

In the GBD 2021 study, OA was categorized using International Classification of Diseases, 10th Edition code B.11.2, encompassing hand, hip, knee, and other subtypes. Cases of OA are identified based on clinical symptoms and radiologic confirmation using Kellgren–Lawrence (K–L) grading system levels II–IV (19, 20). DALYs, a key epidemiological metric, measure the burden of disease in terms of the total time lived with disability by both survivors and those who have died. The metric combines premature mortality (YLLs) and disability duration (YLDs), calculated as their sum (DALYs = YLLs + YLDs) (21). Since deaths directly attributed to OA were not reported in GBD 2021, YLLs are assumed to be zero, making DALYs equivalent to YLDs. Consequently, this study utilizes DALYs to evaluate the impact of OA. High BMI (defined as >20–25 kg/m^2^ in adults ≥20 years) is a GBD-validated risk factor for OA, with biomechanical stress explaining its stronger association with weight-bearing joints such as the knee and hip (9).

### 2.3 Statistical analysis

Using GBD 2021 data, this study assessed the OA burden in China, the US, and globally through incidence, prevalence, DALYs, age-standardized incidence rate (ASIR), prevalence rate (ASPR), and disability-adjusted life years (AS-DALYs). All estimates include age-standardized and mean values with 95% uncertainty intervals (UIs) (8). The Joinpoint regression model is an epidemiological tool used for trend analysis. It begins with a minimal number of joinpoint, testing their statistical significance and connecting those with similar trends into a smooth line (22). This model is applicable for analyzing temporal trends in ASIR, ASPR, and AS-DALYs of diseases (23). This study calculated annual percentage change and average annual percentage changes (AAPC) for ASPR, ASIR, and AS-DALY of OA in China, the US, and globally. Annual percentage change reflects changes over specific periods, while AAPC summarizes overall trends. An annual percentage change > 0 indicates an increase, < 0 indicates a decrease, and a value equal to 0 suggests a stable or non-significant change over time.

The APC model is commonly applied in epidemiological research, especially for analyzing chronic non-communicable diseases and cancer trends (24). This model disentangles the effects of age, period, and birth cohort in epidemiological data, enabling a more precise estimation of their independent impacts on disease incidence or mortality. It also provides insights into long-term trends in disease progression over time. This study employed APC analysis to assess OA incidence trends in China, the US, and globally, using 5-year aggregated data (1992–2021) stratified by age. The APC Web Tool was utilized to construct models and derive age-specific rate ratios (RRs) for different periods and cohorts (25).

According to GBD 2021, elevated BMI (≥25 kg/m^2^)—the sole identified risk factor for OA, hip OA, and knee OA—was analyzed due to its causal association with OA risk variation, with corresponding AS-DALYs reported. BAPC analysis was applied to predict OA disease burden trends from 2022 to 2030, using ASIR, ASPR, and AS-DALYs population data, and projected population figures. Statistical analyses and visualizations were performed with R software (v4.4.2) and IHME’s interactive data visualization tools, adopting a significant threshold of *p* < 0.05.

## 3 Results

### 3.1 Disease burden of osteoarthritis among adults aged 55 years and older across global regions in 2021

**Figure 1:**
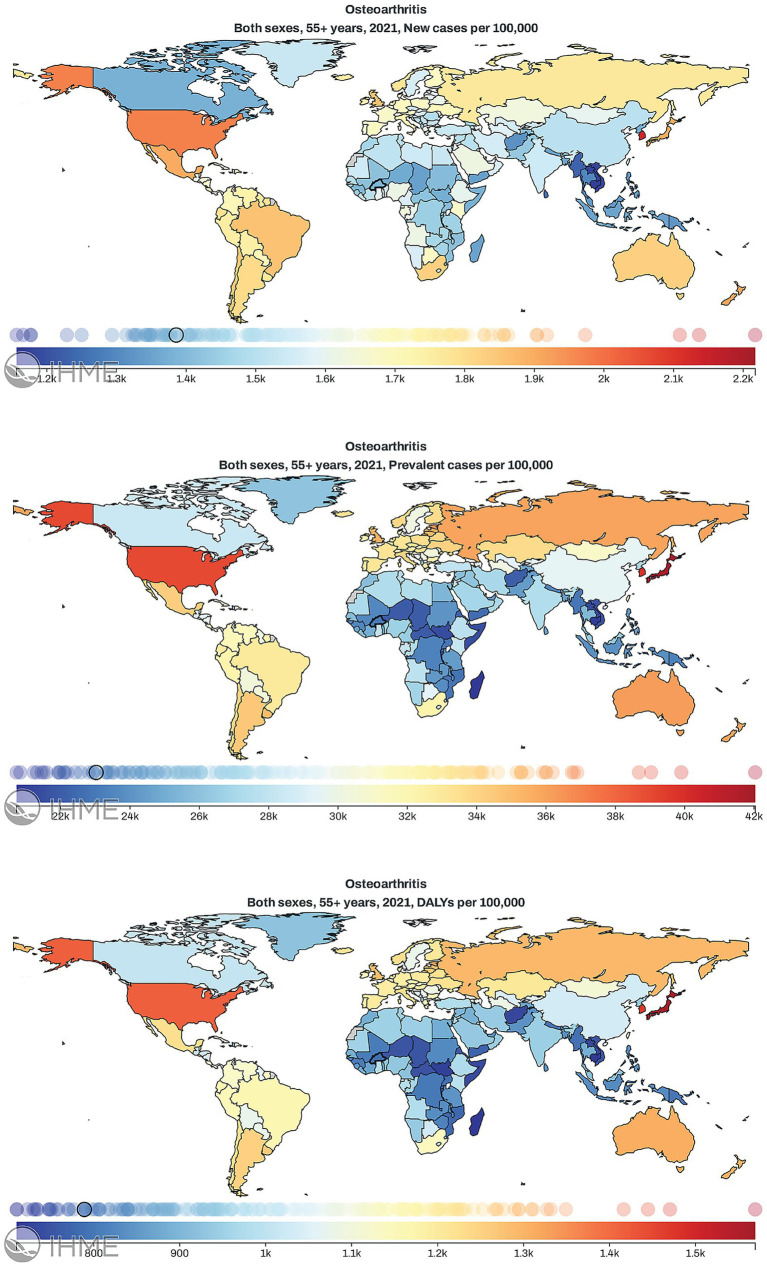
Geographical distribution of the incidence, prevalence, and disability-adjusted life years (DALYs) rates of osteoarthritis in individuals aged 55 years and older worldwide in 2021.

According to the analysis of GBD 2021 data, the burden of OA among individuals aged 55 years and older shows considerable variation across countries (Figure 1; Supplementary Table S1). Figure 1 presents the global distribution of OA incidence, prevalence, and DALYs rates across world regions, generated using IHME’s interactive data visualization tools. Among all countries, the highest levels were consistently observed in the US, Japan, and South Korea. Specifically, in 2021, the US had an incidence rate of 1973.19 per 100,000, a prevalence rate of 39030.76 per 100,000, and a DALYs rate of 1416.48 per 100,000, whereas China showed comparatively lower rates of 1507.57, 29525.33, and 1033.44 per 100,000, respectively. Other G20 countries, including India, Brazil, and South Africa, generally exhibited lower rates across these indicators.

### 3.2 Changes and trends in the disease burden of OA in China and the US

From 1990 to 2021, the ASIR, ASPR, and AS-DALYs of OA increased in both China and the US among individuals aged 55 and older; however, China showed a faster growth rate across all indicators, as shown in Table 1. In China, ASIR of OA increased from 1311.06 (95% UI: 983.94, 1696.27) to 1497.13 (95% UI: 1239.56, 1920.49) per 100,000 population from 1990 to 2021, reflecting an AAPC of 0.46% (95% CI: 0.41, 0.50%). This contrasts with the US, where the ASIR showed a more modest increase from 1878.80 (95% UI: 1432.06, 2386.38) to 2012.44 (95% UI: 1545.37, 2546.84) per 100,000 population over the same period, with an AAPC of 0.20% (95% CI: 0.15, 0.25%). This suggests a faster increase in new OA cases in China compared to the US. Similarly, China experienced a substantial increase in the ASPR, rising from 26227.10 (95% UI: 22733.70, 29936.11) to 30035.65 (95% UI: 26182.66, 34173.46) per 100,000 population with an AAPC of 0.46% (95% CI: 0.44, 0.48%). In contrast, the ASPR in the US showed a smaller increase from 36360.90 (95% UI, 32012.11, 40895.85) to 38266.55 (95% UI, 33761.37, 42953.64) per 100,000 population (AAPC = 0.16%; 95% CI: 0.13, 0.19%). This indicates a higher overall prevalence rate of OA in the US, but a faster growth in prevalence in China. The trend in AS-DALYs followed a similar pattern. China experienced a larger increase in AS-DALYs, from 899.55 (95% UI, 430.98, 1813.47) to 1050.35 (95% UI, 502.60, 2117.86) per 100,000 population, with an AAPC of 0.52% (95% CI, 0.50, 0.54%). In comparison, AS-DALYs in the US increased from 1322.63 (95% UI, 646.60, 2667.35) to 1387.79 (95% UI, 681.85, 2793.00) per 100,000 population (AAPC = 0.15%; 95% CI: 0.12, 0.18%). In addition to age-standardized rates, the absolute number of OA cases has grown substantially, particularly in China. According to Table 2, the number of incident cases in China rose dramatically from 1.90 million in 1990 to 5.71 million in 2021, and prevalent cases expanded from 36.1 million to 111.9 million. In comparison, the US experienced a more modest increase from 0.95 million to 1.98 million incident cases, and from 19.7 million to 39.1 million prevalent cases. These findings highlight distinct epidemiological patterns: China’s massive population generates larger absolute OA burden, though with currently lower per-capita rates than the US. The accelerating growth in China’s case numbers and age-standardized rates signals an emerging public health challenge, while the US maintains persistently high prevalence. These disparities may stem from fundamental differences in population demographics, risk factor profiles (particularly obesity and aging patterns), and healthcare systems between the two countries.

**Table 1:** Global and regional AAPCs of ASR for osteoarthritis among population aged 55 and above (1990–2021) years for osteoarthritis in China, the US, and globally from 2022 to 2030.

Measures	Joint Site	Location	1990 ASR per 100,000 (95 %UI)	2021 ASR per 100,000 (95 %UI)	AAPC (%) (95% CI)
Incidence	OA	China	1311.06 (983.94, 1696.27)	1497.13 (1129.56, 1920.49)	0.46 (0.41, 0.5)
		USA	1878.80 (1432.06, 2386.38)	2012.44 (1545.37, 2546.84)	0.2 (0.15, 0.25)
		Global	1479.08 (1133.77, 1875.74)	1595.66 (1219.74, 2018.12)	0.24 (0.23, 0.24)
	Hip OA	China	36.11 (18.05, 62.80)	47.70 (24.02, 82.78)	0.91 (0.89, 0.93)
		USA	120.59 (65.03, 196.12)	140.00 (75.65, 223.70)	0.38 (0.23, 0.51)
		Global	65.49 (34.74, 108.94)	69.77 (36.87, 116.81)	0.19 (0.17, 0.21)
	Knee OA	China	932.72 (661.99, 1299.13)	1007.33 (717.55, 1391.49)	0.31 (0.27, 0.35)
		USA	1181.98 (836.57, 1622.35)	1265.01 (901.48, 1731.27)	0.21 (0.16, 0.26)
		Global	985.68 (711.42, 1341.28)	1046.80 (754.62, 1420.43)	0.19 (0.19, 0.2)
	Hand OA	China	208.70 (104.54, 359.81)	305.02 (158.05, 520.07)	1.22 (1.18, 1.25)
		USA	450.17 (241.54, 755.21)	479.25 (260.63, 803.49)	0.16 (0.11, 0.22)
		Global	296.13 (154.51, 504.48)	344.20 (182.25, 584.08)	0.48 (0.46, 0.5)
	Other OA	China	133.53 (75.90, 196.18)	137.09 (78.29, 201.32)	0.08 (0.08, 0.08)
		USA	126.06 (72.20, 179.75)	128.18 (73.30, 184.04)	0.05 (0.05, 0.05)
		Global	131.78 (74.67, 192.77)	134.89 (76.50, 197.73)	0.08 (0.07, 0.08)
Prevalence	OA	China	26227.10 (22733.70, 29936.11)	30035.65 (26182.66, 34173.46)	0.46 (0.44, 0.48)
		USA	36360.90 (32012.11, 40895.85)	38266.55 (33761.37, 42953.64)	0.16 (0.13, 0.19)
		Global	28414.43 (24919.20, 32042.51)	30780.85 (26958.70, 34687.41)	0.26 (0.26, 0.26)
	Hip OA	China	861.64 (629.92, 1152.82)	1127.83 (822.32, 1506.74)	0.88 (0.86, 0.9)
		USA	3774.23 (2807.14, 4927.92)	4255.14 (3167.19, 5585.15)	0.35 (0.2, 0.46)
		Global	1792.88 (1338.09, 2339.65)	1884.41 (1399.51, 2471.63)	0.15 (0.13, 0.17)
	Knee OA	China	19372.92 (16062.84, 23170.79)	20818.38 (17286.02, 24842.32)	0.24 (0.22, 0.27)
		USA	20981.45 (17418.65, 25112.13)	21977.65 (18285.47, 26301.54)	0.21 (0.16, 0.27)
		Global	17359.32 (14411.50, 20747.72)	18716.67 (15561.72, 22294.81)	0.25 (0.24, 0.25)
	Hand OA	China	4764.73 (3336.04, 6496.44)	7643.31 (5469.27, 10267.35)	1.6 (1.53, 1.66)
		USA	14005.49 (10177.85, 18312.69)	15031.10 (10911.08, 19727.95)	0.2 (0.14, 0.26)
		Global	9225.55 (6718.98, 12113.15)	10549.31 (7673.08, 13849.38)	0.42 (0.41, 0.44)
	Other OA	China	3229.56 (2418.28, 4247.19)	3439.40 (2582.30, 4535.64)	0.2 (0.2, 0.21)
		USA	3221.37 (2606.32, 3933.20)	3420.31 (2772.63, 4174.48)	0.19 (0.19, 0.19)
		Global	3243.35 (2454.28, 4238.10)	3420.08 (2585.38, 4478.75)	0.17 (0.17, 0.17)
DALYs	OA	China	899.55 (430.98, 1813.47)	1050.35 (502.60, 2117.86)	0.52 (0.5, 0.54)
		USA	1322.63 (646.60, 2667.35)	1387.79 (681.85, 2793.00)	0.15 (0.12, 0.18)
		Global	997.37 (485.70, 2012.81)	1089.13 (529.33, 2195.82)	0.28 (0.28, 0.29)
	Hip OA	China	27.26 (12.55, 55.43)	35.57 (16.40, 72.42)	0.87 (0.85, 0.89)
		USA	118.57 (56.17, 238.79)	131.69 (62.19, 264.87)	0.27 (0.11, 0.39)
		Global	56.15 (26.41, 113.07)	58.91 (27.67, 118.85)	0.15 (0.12, 0.17)
	Knee OA	China	619.15 (296.95, 1216.47)	664.23 (318.29, 1306.15)	0.23 (0.21, 0.25)
		USA	662.72 (317.17, 1308.71)	684.59 (329.59, 1349.89)	0.16 (0.11, 0.21)
		Global	549.68 (263.64, 1083.29)	591.87 (283.90, 1164.68)	0.24 (0.24, 0.25)
	Hand OA	China	150.53 (67.38, 313.30)	241.47 (109.25, 501.00)	1.59 (1.53, 1.65)
		USA	440.37 (199.58, 907.53)	465.79 (211.90, 960.78)	0.15 (0.1, 0.21)
		Global	289.48 (131.89, 594.84)	330.77 (150.61, 681.54)	0.42 (0.4, 0.43)
	Other OA	China	102.60 (46.35, 220.24)	109.09 (49.74, 234.11)	0.2 (0.19, 0.2)
		USA	100.97 (48.38, 211.51)	105.72 (51.02, 221.78)	0.15 (0.14, 0.15)
		Global	102.06 (46.57, 218.10)	107.58 (49.21, 230.09)	0.17 (0.17, 0.17)

AAPC, average annual percentage change; ASRs, Age-standardized rates; UI, uncertainty interval; CI, Confidence interval; OA, Osteoarthritis; DALYs, Disability-adjusted life-years.

**Table 2:** The number and rate of incidence, prevalence, and DALYs in 1990 and 2021 for osteoarthritis among individuals aged 55 years and older in China, the US and globally.

Measures	Location	1990 Number ×10^3^ (95% UI)	1990 rate per 100,000 (95% UI)	2021 Number ×10^3^ (95% UI)	2021 Rate per 100,000 (95% UI)
Incidence	China	1900.81 (1590.01, 2266.24)	1324.44 (1107.88, 1579.06)	5713.02 (4878.67, 6689.21)	1507.57 (1287.40, 1765.17)
	US	954.37 (820.86, 1112.05)	1819.17 (1564.66, 2119.72)	1978.07 (1695.69, 2308.44)	1973.19 (1691.50, 2302.74)
	Global	10099.03 (8568.10, 11803.21)	1504.12 (1276.11, 1757.93)	23858.14 (20407.94, 27685.71)	1605.54 (1373.36, 1863.12)
Prevalence	China	36116.01 (31771.06, 40575.77)	25164.79 (22137.33, 28272.25)	111887.74 (98363.58, 125100.38)	29525.33 (25956.52, 33011.92)
	US	19671.77 (17566.09, 21800.35)	37497.03 (33483.32, 41554.37)	39127.46 (34932.84, 43334.90)	39030.76 (34846.51, 43227.80)
	Global	185881.36 (164341.33, 207273.10)	27684.58 (24476.48, 30870.60)	453562.91 (400659.71, 505177.61)	30522.69 (26962.55, 33996.12)
DALYs	China	1241.20 (597.76, 2497.07)	864.84 (416.50, 1739.90)	3916.28 (1879.72, 7933.21)	1033.44 (496.03, 2093.44)
	US	716.78 (350.96, 1457.81)	1366.27 (668.99, 2778.79)	1419.99 (702.11, 2879.60)	1416.48 (700.38, 2872.48)
	Global	6525.96 (3164.70, 13190.23)	971.96 (471.34, 1964.51)	16050.20 (7768.33, 32489.95)	1080.10 (522.77, 2186.42)

Rate, Age-specific rate; DALYs, disability-adjusted life years.

### 3.3 Rapidly increasing burden of hand and hip osteoarthritis in China compared to other subtypes and the US

In China, the AAPCs for hand and hip OA ASPR from 1990 to 2021 are 1.6% (95% CI: 1.53–1.66) and 0.88% (95% CI: 0.86–0.90), respectively (Table 1). These values are higher than the AAPCs for overall OA (0.46, 95% CI: 0.44–0.48) and knee OA (0.24, 95% CI: 0.22–0.27). This indicates a sharper increase in the burden of hand and hip OA compared to other OA subtypes in China. For ASIR, hand OA and hip OA in China also show a relatively higher AAPC. Hand OA has an AAPC of 1.22% (95% CI: 1.18–1.25), and hip OA stands at 0.91% (95% CI: 0.89–0.93), surpassing the overall OA AAPC of 0.46% (95% CI: 0.41–0.50). This further highlights the rapid growth of new cases in these specific OA subtypes. In terms of AS-DALYs, the AAPC for hand OA is 1.59% (95% CI: 1.53–1.65), and for hip OA, it is 0.87% (95% CI: 0.85–0.89). These rates are much higher compared to the AAPC for knee OA (0.23, 95% CI: 0.21–0.25) and overall OA (0.52, 95% CI: 0.50–0.54). This indicates a faster-growing disability burden from hand and hip OA among Chinese adults aged ≥55 years. In contrast, the US shows lower AAPCs across all subtypes of OA. For instance, the AAPCs for hand OA ASPR is 0.2% (95% CI: 0.14–0.26), and for hip OA, it is 0.35% (95% CI: 0.20–0.46). Similarly, ASIR and AS-DALYs for hand and hip OA have AAPCs below 0.4%, much lower than China’s values. The AAPCs for hand and hip OA in China are remarkably higher than those for other OA subtypes in the country and significantly exceed the corresponding rates in the US. These findings indicate a need for targeted public health interventions in China, focusing particularly on the rapidly increasing burden of hand and hip OA.

### 3.4 Temporal and sex-specific patterns of osteoarthritis burden in China, the US, and globally

**Figure 2:**
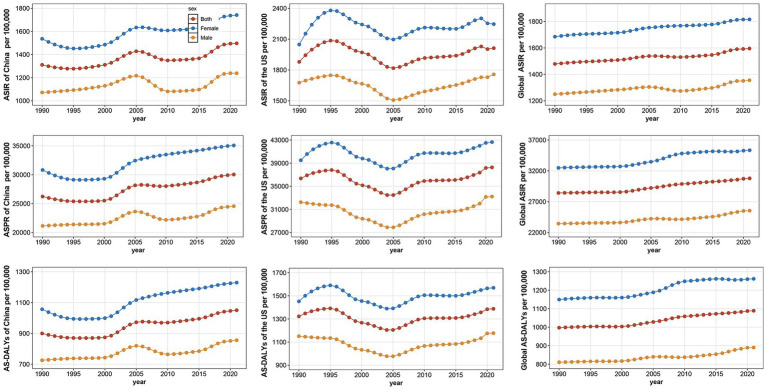
Trends in the age-standardized incidence rate (ASIR), prevalence rate (ASPR), and disability-adjusted life years (AS-DALYs) per 100,000 population for osteoarthritis by sex in China, the United States, and globally from 1990 to 2021.

Regarding the ASIR of OA in individuals aged 55 and older, China exhibited an overall upward trajectory, characterized by a sharp rise between 2000 and 2005, followed by a gradual decline until 2015 and a subsequent slow rising (Figure 2). In the US, the ASIR rose rapidly from 1990 to 1995, peaked in 1995, and then slowly declined, reaching a trough in 2005 before rising again gradually (Figure 2). In China, the US, and globally, the trends in ASPR and AS-DALYs mirror those observed for ASIR. Furthermore, in both countries, all three indicators for OA are generally higher in females than in males.

### 3.5 Age-specific disease burden of different osteoarthritis subtypes among individuals aged 55 years and older in China, the US, and globally in 2021

**Figure 3:**
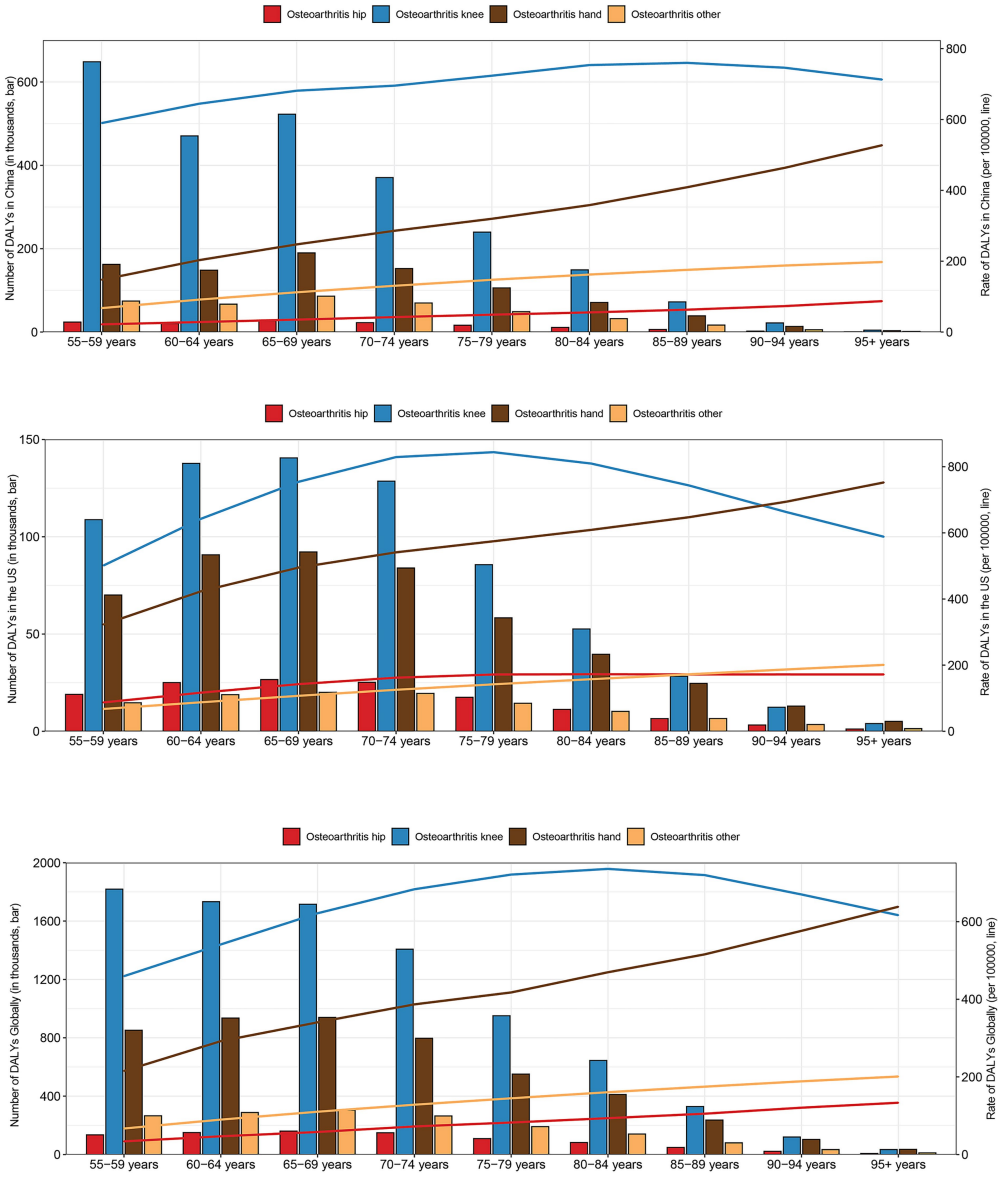
The number and rate of disability-adjusted life years (DALYs) due to osteoarthritis of the hip, knee, hand, and other sites by age group in China (top), the United States (US) (middle), and globally (bottom) in 2021.

As shown in Figure 3, together with Supplementary Figures S1, S2, the age-specific distribution of OA burden among individuals aged 55 years and older was analyzed by both number and rate of DALYs, incidence and prevalence across different joint sites (hip, knee, hand, and other OA) in China, the US, and globally. In China, the largest DALYs number for knee OA was observed in the 55–59 years age group. However, the DALYs rate for knee OA peaked in the 85–89 years age group, indicating a shift in the age-related impact from absolute counts to relative rates. For hand OA in China, the largest DALYs number was observed in the 65–69 years group, while its rate continued to increase with advancing age, becoming especially prominent in the oldest age groups. In the US, the highest number of DALYs from knee OA occurred in the 65–69 years group, while the DALYs rate peaked in the 75–79 years group. In the US, the proportion of hand OA-related DALYs rate was consistently higher than in China across all age groups. In both countries, hand OA-related DALYs rate increased steadily with age, and the absolute number of DALYs peaked around 65–69 years. Hip OA in both China and the US contributed a smaller proportion of total DALYs, but its rate gradually increased with age, reflecting the cumulative burden in the oldest individuals. Similarly, across all regions, the rate of DALYs due to “other OA” types showed a consistent rise with age, becoming a more significant contributor among those aged 90 years and older. Overall, knee OA remains the leading contributor to OA-related DALYs in younger older adults, while hand OA and other OA become increasingly important in the oldest age groups. These findings highlight both joint-specific and age-specific patterns of OA burden, with noticeable differences between China and the US.

### 3.6 Age-period-cohort analysis for incidence of osteoarthritis in China, the US and globally from 1992 to 2021

**Figure 4:**
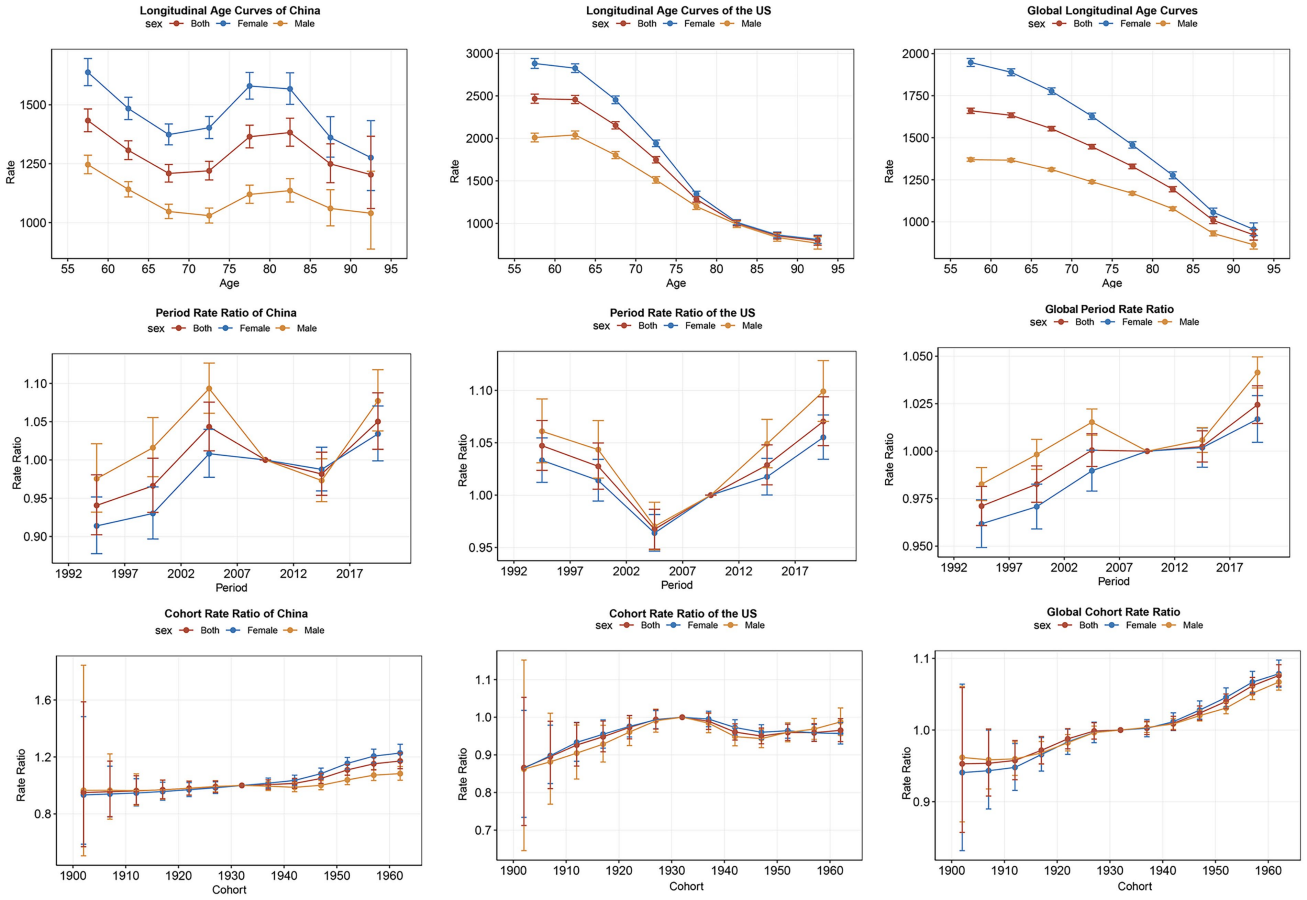
Age-period-cohort models for incidence of osteoarthritis in China, the United States and Globally from 1992 to 2021.

Longitudinal age curves primarily describe the age effect, illustrating the trend of disease incidence as age increases (Figure 4). The analysis of age effects reveals that globally and, in the US, the incidence of OA gradually decreases with increasing age. However, in China, there is a significant peak in incidence between the ages of 75 and 85. To illustrate the period effect on OA incidence from 1992 to 2021, we present a comparative analysis across three regions: China, the US, and globally (Figure 4). The period from 2007 to 2012 serves as the reference, with an incidence rate ratio of 1. Distinct patterns emerge across the three regions. In China, incidence gradually increased from 1992, peaking between 2002 and 2007, followed by a decline and a subsequent rebound starting in the 2012–2017 period, which continues to rise. Conversely, the US exhibits a decreasing trend from 1990, reaching its lowest point between 2002 and 2007 before rising again. Globally, a steady increase in incidence is observed. The impact of cohort effects on OA incidence from 1992 to 2021 is analyzed across three regions: China, the US, and globally (Figure 4). This ratio measures the relative risk of developing OA for a specific birth cohort compared to a reference cohort, revealing the long-term influence of birth cohort on incidence. The cohort of individuals born from 1930 to 1935 serves as the reference in this analysis. A general upward trend in OA incidence is observed across birth cohorts from 1905 to 1965 in China, the US, and globally. However, a distinct pattern emerges in the US, where a peak incidence is observed in the cohort born in the 1930s, followed by a slight decline and a subsequent gradual increase from the 1940s onwards.

### 3.7 Risk factor analysis of BMI contribution to the burden of hip and knee osteoarthritis in China, the US, and globally

We analyzed the contribution of BMI to the AS-DALYs associated with hip and knee OA in individuals aged 55 and older (Table 3). For hip OA, China exhibited a substantial rise in AS-DALYs, escalating from 4.47 (−0.34, 12.50) per 100,000 population in 1990 to 10.39 (−0.86, 28.38) in 2021, accompanied by an AAPC of 2.76 (2.74, 2.79). This marked growth in disability burden was also observed in the US, albeit less pronounced, with AS-DALYs increasing from 46.41 (−4.56, 127.95) in 1990 to 60.56 (−6.59, 155.96) in 2021 and an AAPC of 0.78 (0.64, 0.88). Globally, the trend mirrored that of the US, with AS-DALYs rising from 16.78 (−1.48, 45.45) in 1990 to 20.99 (−1.98, 55.41) in 2021 and an AAPC of 0.73 (0.7, 0.75). For knee OA, China presented that AS-DALYs increased substantially from 105.50 (−8.48, 314.53) in 1990 to 202.36 (−17.87, 580.46) in 2021, with an AAPC of 2.13 (2.11, 2.15). In the US, the values rose from 261.06 (−25.32, 706.24) in 1990 to 316.51 (−34.78, 837.30) in 2021, with an AAPC of 0.68 (0.63, 0.73), while globally, they climbed from 142.68 (−12.32, 405.07) in 1990 to 197.16 (−18.42, 549.13) in 2021, with an AAPC of 1.05 (1.05, 1.06). While the US exhibits higher AS-DALYs values for hip and knee OA, China demonstrates a more rapid growth rate in AS-DALYs, indicating that the contribution of BMI to the escalating burden of OA is increasing faster in China compared to the US. This observation suggests that while BMI currently plays a more prominent role in the OA burden in the US, its impact is accelerating more rapidly in China.

**Table 3:** Global and regional AS-DALYs of hip and knee osteoarthritis associated with high body mass index.

Joint site	Location	1990 AS-DALYs per 100,000 (95 %UI)	2021 AS-DALYs per 100,000 (95 %UI)	AAPC (%) (95% CI)
Hip OA	China	4.47 (−0.34, 12.50)	10.39 (−0.86, 28.38)	2.76 (2.74, 2.79)
	USA	46.41 (−4.56, 127.95)	60.56 (−6.59, 155.96)	0.78 (0.64, 0.88)
	Global	16.78 (−1.48, 45.45)	20.99 (−1.98, 55.41)	0.73 (0.7, 0.75)
Knee OA	China	105.50 (−8.48, 314.53)	202.36 (−17.87, 580.46)	2.13 (2.11, 2.15)
	USA	261.06 (−25.32, 706.24)	316.51 (−34.78, 837.30)	0.68 (0.63, 0.73)
	Global	142.68 (−12.32, 405.07)	197.16 (−18.42, 549.13)	1.05 (1.05, 1.06)

AS-DALYs, Age-standardized rates of Disability-adjusted life-years; AAPC, average annual percentage change; UI, uncertainty interval; CI, confidence interval; OA, osteoarthristis.

### 3.8 Projections of the osteoarthritis burden in China, the US, and globally to 2030

**Figure 5:**
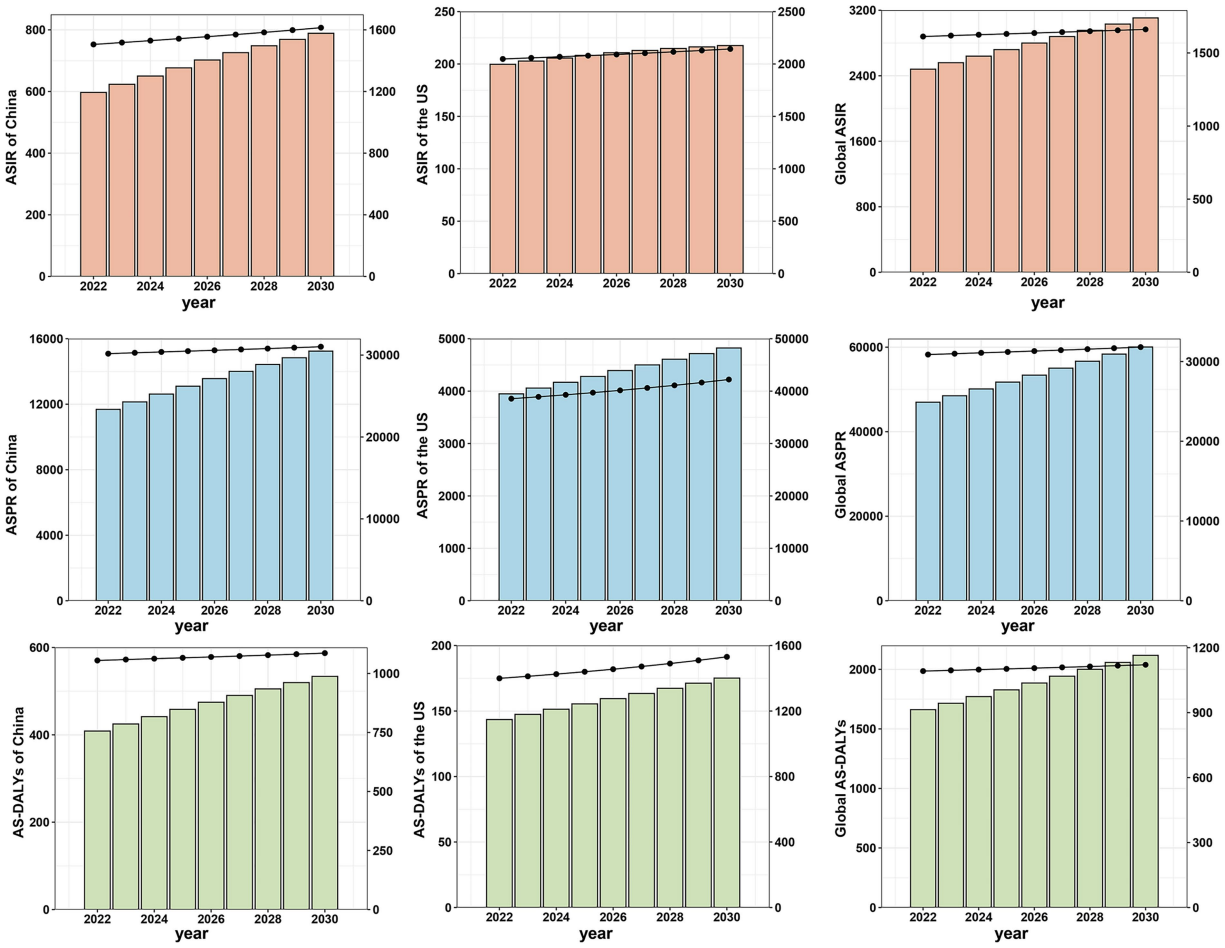
Projections of age-standardized incidence, prevalence, and disability-adjusted life.

To project the burden of OA in China, the US, and globally, from 2022 to 2030, we employed the BAPC prediction model to estimate ASIR, ASPR, and AS-DALYs in individuals aged 55 and older. Figure 5 presents the model’s projections, with the left y-axis showing the number of cases and the right y-axis displays the ASR data. The projections indicate a persistent rise in OA burden across China, the US, and globally through 2030. While China currently exhibits lower ASIR, ASPR, and AS-DALYs than the US, it demonstrates a higher rate of increase. Taking ASIR as an example, projections indicate that China will experience a substantial increase, from 1,505 in 2022 to 1,614 in 2030 per 100,000 population, representing a growth rate of about 7.2%. In contrast, the US is projected to see a more modest rise from 2048 to 2,143 per 100,000 population, with a growth rate of approximately 4.6%. Globally, ASIR is expected to rise from 1,612 to 1,661 per 100,000 population, reflecting a growth rate of about 3.0%. Projections indicate a persistent increase in the OA burden among individuals aged 55 and older in China, the US, and globally through 2030. While China currently exhibits lower ASIR, ASPR, and AS-DALYs than the US, it demonstrates a higher rate of increase. This faster growth in China suggests that public health interventions and healthcare resources may need to be scaled up more rapidly in China compared to the US to address the future burden of OA effectively.

## 4 Discussion

This study offers a thorough examination of OA burden among adults aged ≥55 years in China, the US, and globally from 1990 to 2021, with a focus on site-specific OA. Our findings indicate that although the age-specific and age-standardized rates of OA burden are consistently higher in the US compared to China, the growth trend is more moderate in the US. In contrast, China, due to its large population base, exhibits substantially higher absolute numbers of cases, despite lower rates, and is experiencing a more rapid escalation in the overall burden. Notably, hand and hip OA in China showed the highest growth rates (AAPC = 1.6 and 0.88%, respectively), significantly outpacing knee OA (AAPC = 0.24%). Significant gender disparities were observed, with females exhibiting a consistently higher OA burden across all regions. High BMI is a recognized risk factor for hip and knee OA, and our findings suggest that its associated burden may be increasing more rapidly in China. Our findings align with the WHO Healthy Aging Framework in demonstrating the urgent need for population-level interventions that preserve mobility and reduce disability. The rapid increase in OA burden in China—driven by rapid urbanization, dietary transitions, and reduced physical activity, particularly in weight-bearing and hand joints—may compromise functional ability among older adults, especially in the absence of widespread preventive measures (26, 27). Meanwhile, the persistently high OA burden in the US—fueled by entrenched individual-level risk factors like obesity—suggests a continued challenge in achieving the WHO’s goal of maintaining functional ability despite advanced health infrastructure (28). These findings highlight the need for interventions that address both individual behaviors, such as weight management and physical activity, and broader structural factors, including urban planning and healthcare access (29, 30). Integrating OA prevention and management into aging policies is essential to promote equitable functional health outcomes for older adults in both high- and middle-income countries.

The ASIR of OA among adults aged ≥55 shows distinct temporal trends in China and the US, shaped by their differing demographic and socioeconomic contexts. In China, ASIR rose sharply between 2000–2005, declined until 2015, and then increased gradually. The US peaked earlier in 1995, followed by a decline and steady rise. These shifts likely reflect changes in lifestyle, aging patterns, and healthcare improvements. ASPR and AS-DALYs followed similar patterns, with women consistently showing higher values—likely due to hormonal, anatomical, and longevity factors (31). These gender disparities and national differences highlight the need for adaptive healthcare strategies, including early intervention and weight management (32). Further research should assess the effects of targeted interventions and social determinants to improve OA prevention in older adults (33). Our age-specific analysis highlights a clear shift in OA burden across age groups and joint sites. Knee OA dominates the burden in younger older adults (55–69 years), while hand OA and other OA types contribute more prominently to disability in the oldest age groups (≥80 years), especially in China. This aligns with previous studies showing age-related changes in joint vulnerability (8). The rising disability burden rate for hand OA in advanced age, particularly in China, suggests an overlooked contributor to late-life disability. These findings emphasize the need for OA management strategies that not only target weight-bearing joints but also address hand function preservation in aging populations.

The analysis of longitudinal age curves highlights distinct OA incidence patterns. While OA incidence globally and in the US decreases with age, China shows a significant peak between ages 75 and 85, likely driven by its rapidly aging population and increasing life expectancy (34). This emphasizes the need for targeted interventions in China’s older adults (35). Period effects reveal temporal differences, with China experiencing a peak between 2002 and 2007, a decline, and a rebound after 2012. This pattern may reflect healthcare reforms and lifestyle shifts. In contrast, the US showed a decline until 2007, followed by a gradual rise, possibly due to sustained public health efforts and improved diagnostic practices (36). Cohort analysis shows an upward trend in OA risk for individuals born between 1905 and 1965, reflecting increased exposure to risk factors like obesity. A distinct peak in the 1930s cohort in the US suggests generational differences influenced by advancements in public health. These findings underscore the importance of age-tailored and cohort-specific approaches to address the increasing OA burden. BMI’s role in OA burden was particularly pronounced for hip and knee OA. The higher AS-DALYs of hip OA and knee OA in the US, which exceed those observed in China and at the global level, may suggest that BMI plays a relatively more substantial role in the burden of hip and knee OA in the US (13). However, the AS-DALYs attributable to BMI for hip OA and knee OA in China showed a higher AAPC of 2.76 and 2.13% respectively, far exceeding the US rate of 0.78 and 0.68%. These results underscore the importance of public health interventions addressing weight management, particularly in rapidly urbanizing regions (37). The BAPC model predicts a continued rise in OA burden among adults aged ≥55 in China, the US, and globally through 2030. Although China currently has lower rates than the US, it is expected to experience the most rapid increase, driven by aging, urbanization, and rising obesity. While the US shows slower projected growth—likely due to established systems and preventive efforts—it still faces significant challenges, especially among older adults with comorbidities.

The study’s findings reveal a notably faster increase in hand and hip OA burden among older adults in China compared to other joint types. This trend underscores the urgent need for enhanced public health responses at the primary care level. Early OA screening programs should be implemented in community health centers, particularly targeting adults aged 55 and above who are at high risk for hand and hip OA. These programs can facilitate timely diagnosis and intervention, potentially mitigating long-term disability (38). Furthermore, the growing influence of high BMI on OA—especially in weight-bearing joints like the hip and knee—suggests that weight management should be prioritized in national aging health strategies (27). This aligns with the WHO’s Global Strategy and Action Plan on Ageing and Health, which advocates integrating chronic disease prevention into aging care systems. In fact, “weight management” emerged as a key topic during China’s 2025 Two Sessions, reflecting rising governmental and public interest (39). The National Health Commission’s three-year initiative, titled “Year of Weight Management,” prioritizes community-level weight control programs, which may substantially decrease joint loading forces in weight-bearing joints, especially for aging populations. Tailored weight management programs for older adults, public education campaigns, and health promotion activities—such as lectures on OA risk factors and BMI control—can be delivered through community health platforms. These initiatives will foster early awareness, encourage healthy lifestyles, and reduce OA-related disability among China’s rapidly aging population.

While the US continues to exhibit higher absolute levels of OA incidence and disability, the relatively slower rate of increase suggests some effectiveness of early detection and chronic disease management programs. However, our analysis shows that BMI remains a major contributor to OA-related disability, particularly among older women and those with hip or knee involvement. These findings suggest the multifactorial nature of female predominance in OA, involving complex interactions between menopausal hormonal changes, and sex-specific musculoskeletal anatomy, as evidenced by recent meta-analyses of sex-specific risk factors (40). Future public health strategies in the US should focus on expanding OA management into broader chronic care models. This includes integrating OA screening and weight control programs into Medicare and Medicaid reimbursement policies and promoting non-pharmacological interventions—such as physical activity and dietary guidance—for high-risk elderly populations (41). Additionally, a shift toward multidisciplinary, integrated care pathways—especially for aging individuals with multiple comorbidities—may improve long-term outcomes and help reduce healthcare system burdens (42). The findings suggest that even in high-income countries with mature healthcare systems, sustained attention to lifestyle-related risk factors remains essential for effective OA control in the context of aging.

This study has some limitations: First, the GBD 2021 dataset primarily derives from sources such as administrative epidemiological surveillance, census data, and disease registries. The indicators are derived through mathematical modeling rather than dedicated OA population studies, which may result in discrepancies between the estimates and the actual burden. Potential under detection of OA in low-income regions could lead to systematically underestimated prevalence in GBD data. Second, while examining trends in China, the US, and globally, the research does not extend to other territories, potentially limiting its ability to capture regional variations and specific needs. Third, while various risk factors contribute to OA and site-specific OA, including labor intensive jobs, vigorous sports, and history of joint trauma, the GBD 2021 dataset includes only high BMI as a risk factor. This restricts the comprehensiveness of the risk factor analysis, leaving out other potentially significant contributors to OA. Despite these limitations, this study provides valuable multi-level insights and future projections, highlighting the necessity of tailored interventions to mitigate the growing OA burden.

## 5 Conclusion

This study highlights the increasing burden of OA in individuals aged 55 and older in China and the US from 1990 to 2021, with projections through 2030. While the US exhibits higher absolute rates of OA incidence, prevalence, and disability, China shows significantly faster growth, particularly for hand and hip OA demanding urgent preventive strategies to curb accelerating burdens. These findings highlight the critical need for tailored healthcare strategies that address both aging populations and evolving lifestyle factors. By evaluating and comparing the prevalence, patterns, and driving factors of OA in China, the US and globally, this study identifies key disease-influencing elements to inform tailored prevention and management strategies for their unique populations, while providing globally applicable insights to guide preventive approaches and advance clinical practice worldwide.

## Data Availability

The original contributions presented in the study are included in the article/Supplementary material, further inquiries can be directed to the corresponding author.

## Footnotes

^1^ https://www.healthdata.org/research-analysis/gbd
