# Supplementary material for: Burden of osteoarthritis in older adults (aged ≥55 years) in the United States and China: a comparative analysis of temporal trends, risk factor contributions, and projected burden to 2030 using global burden of disease study 2021 data
Source: Front Med (Lausanne). 2025 Sep 16;12:1636976. doi: 10.3389/fmed.2025.1636976 (PMC12481515; doi:10.3389/fmed.2025.1636976)
Supplement: Supplementary file 1 [file Data_Sheet_1.docx]

**Table S1.** The number and rate of incidence, prevalence, and DALYs in 2021 for osteoarthritis among individuals aged 55 years and older across G20 countries and globally.

| Locations | Incidence | | Prevalence | | DALYs | |
| --- | --- | --- | --- | --- | --- | --- |
|  | Number×10^3^  (95% UI) | Rate per100,000 (95% UI) | Number×10^3^  (95% UI) | Rate  per100,000 (95% UI) | Number×10^3^  (95% UI) | Rate per100,000  (95% UI) |
| Argentina | 168.43 (144.37-194.71) | 1804.58 (1546.84-2086.19) | 3231.37 (2891.14-3578.03) | 34621.90 (30976.54-38336.15) | 116.91 (57.15-235.79) | 1252.65 (612.32-2526.35) |
| Australia | 135.43 (116.92-156.90) | 1828.24 (1578.37-2118.07) | 2681.25 (2403.30-2981.78) | 36194.78 (32442.65-40251.75) | 97.12 (47.96-197.33) | 1311.01 (647.42-2663.77) |
| Brazil | 804.03 (684.51-943.10) | 1856.55 (1580.57-2177.66) | 14271.00 (12620.66-15910.57) | 32952.53 (29141.81-36738.38) | 504.14 (246.64-1018.02) | 1164.09 (569.28-2350.66) |
| Canada | 168.98 (144.40-197.64) | 1377.07 (1176.75-1610.62) | 3477.52 (3060.81-3925.69) | 28338.90 (24943.07-31991.16) | 123.29 (60.01-249.34) | 1004.68 (489.05-2031.90) |
| China | 5713.02 (4878.67-6689.21) | 1507.57 (1287.40-1765.17) | 111887.74 (98363.58-125100.38) | 29525.33 (25956.62-33011.92) | 3916.28 (1879.72-7933.21) | 1033.44 (496.03-2093.44) |
| France | 369.29 (319.41-425.16) | 1670.23 (1444.61-1922.92) | 7357.55 (6631.54-8096.16) | 33276.84 (29993.25-36617.44) | 264.18 (129.57-532.12) | 1194.86 (586.04-2406.67) |
| Germany | 534.14 (463.88-615.11) | 1694.93 (1472.00-1951.86) | 10463.81 (9395.20-11617.87) | 33203.77 (29812.86-36865.87) | 375.69 (185.05-756.42) | 1192.14 (587.21-2400.26) |
| India | 3101.45 (2624.65-3642.40) | 1542.66 (1305.50-1811.73) | 55046.10 (48465.22-61669.34) | 27379.94 (24106.61-30674.34) | 1899.78 (923.21-3840.13) | 944.95 (459.20-1910.08) |
| Indonesia | 564.91 (472.15-677.17) | 1348.90 (1127.40-1616.95) | 9980.71 (8669.23-11250.04) | 23831.95 (20700.39-26862.84) | 347.83 (169.83-697.21) | 830.55 (405.53-1664.80) |
| Italy | 393.38 (344.14-452.56) | 1731.69 (1514.93-1992.17) | 7754.51 (6960.21-8552.87) | 34135.59 (30639.05-37649.99) | 279.99 (136.73-570.31) | 1232.53 (601.88-2510.52) |
| Japan | 994.05 (869.23-1135.05) | 1904.29 (1665.15-2174.39) | 21941.72 (19788.20-24159.37) | 42033.21 (37907.76-46281.50) | 819.29 (394.81-1664.39) | 1569.49 (756.34-3188.43) |
| Mexico | 410.21 (346.56-483.82) | 1903.49 (1608.13-2245.07) | 7395.77 (6525.56-8243.70) | 34318.40 (30280.40-38253.03) | 264.53 (130.01-534.25) | 1227.50 (603.26-2479.08) |
| Republic of Korea | 357.32 (304.66-413.89) | 2136.60 (1821.73-2474.88) | 6673.13 (5921.27-7374.00) | 39902.07 (35406.34-44092.94) | 245.90 (118.24-496.63) | 1470.35 (707.04-2969.61) |
| Russian Federation | 751.60 (640.04-875.80) | 1768.26 (1505.78-2060.45) | 15297.95 (13370.05-17318.95) | 35990.75 (31455.07-40745.47) | 550.14 (268.27-1114.53) | 1294.28 (631.15-2622.11) |
| Saudi Arabia | 48.87 (39.86-59.03) | 1623.10 (1324.03-1960.76) | 810.96 (710.97-907.48) | 26935.82 (23614.63-30141.84) | 28.15 (13.88-56.82) | 935.01 (460.91-1887.21) |
| South Africa | 144.21 (121.37-169.82) | 1828.08 (1538.57-2152.75) | 2551.88 (2240.92-2855.91) | 32349.13 (28407.19-36203.17) | 90.12 (44.61-181.20) | 1142.48 (565.52-2296.96) |
| Turkey | 253.55 (215.06-298.95) | 1534.66 (1301.72-1809.52) | 4619.88 (4099.35-5134.03) | 27963.27 (24812.56-31075.30) | 161.37 (79.47-327.98) | 976.74 (481.00-1985.20) |
| UK | 391.74 (341.95-450.80) | 1862.75 (1625.98-2143.58) | 7459.99 (6701.37-8259.03) | 35472.77 (31865.49-39272.27) | 271.19 (132.83-546.35) | 1289.50 (631.64-2597.91) |
| US | 1978.07 (1695.69-2308.44) | 1973.19 (1691.50-2302.74) | 39127.46 (34932.84-43334.90) | 39030.76 (34846.51-43227.80) | 1419.99 (702.11-2879.60) | 1416.48 (700.38-2872.48) |
| Global | 23858.14 (20407.94-27685.71) | 1605.54 (1373.36-1863.12) | 453562.91 (400659.71-505177.61) | 30522.69 (26962.55-33996.12) | 16050.20 (7768.33-32489.95) | 1080.10 (522.77-2186.42) |

Rate: Age-specific rate; UK: United Kingdom; US: United States of America; DALYs: disability-adjusted life years


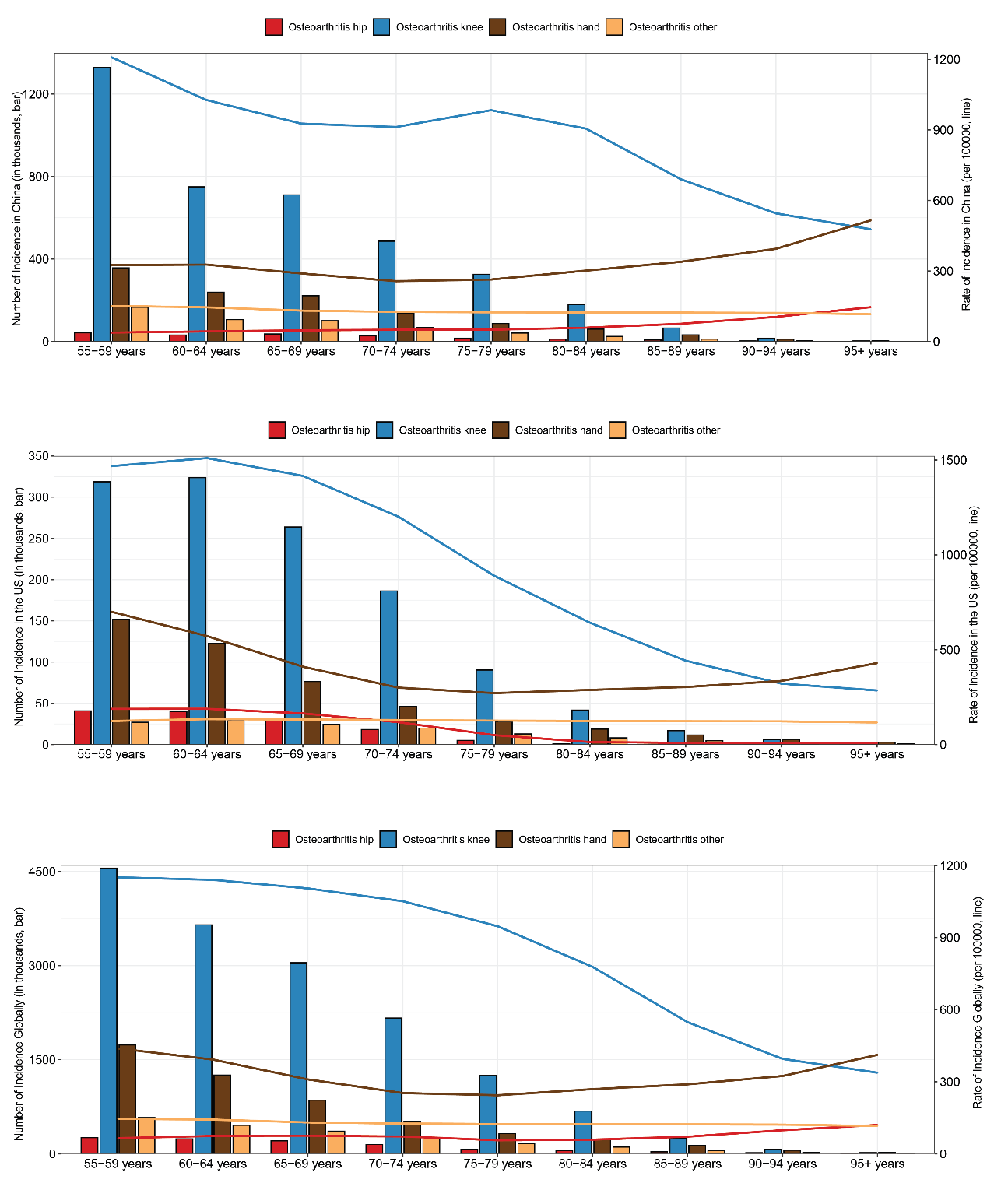
**Figure S1.** The number and rate of incident cases of osteoarthritis (hip, knee, hand, and other sites) by age group in China (top), the United States (middle), and globally (bottom) in 2021.


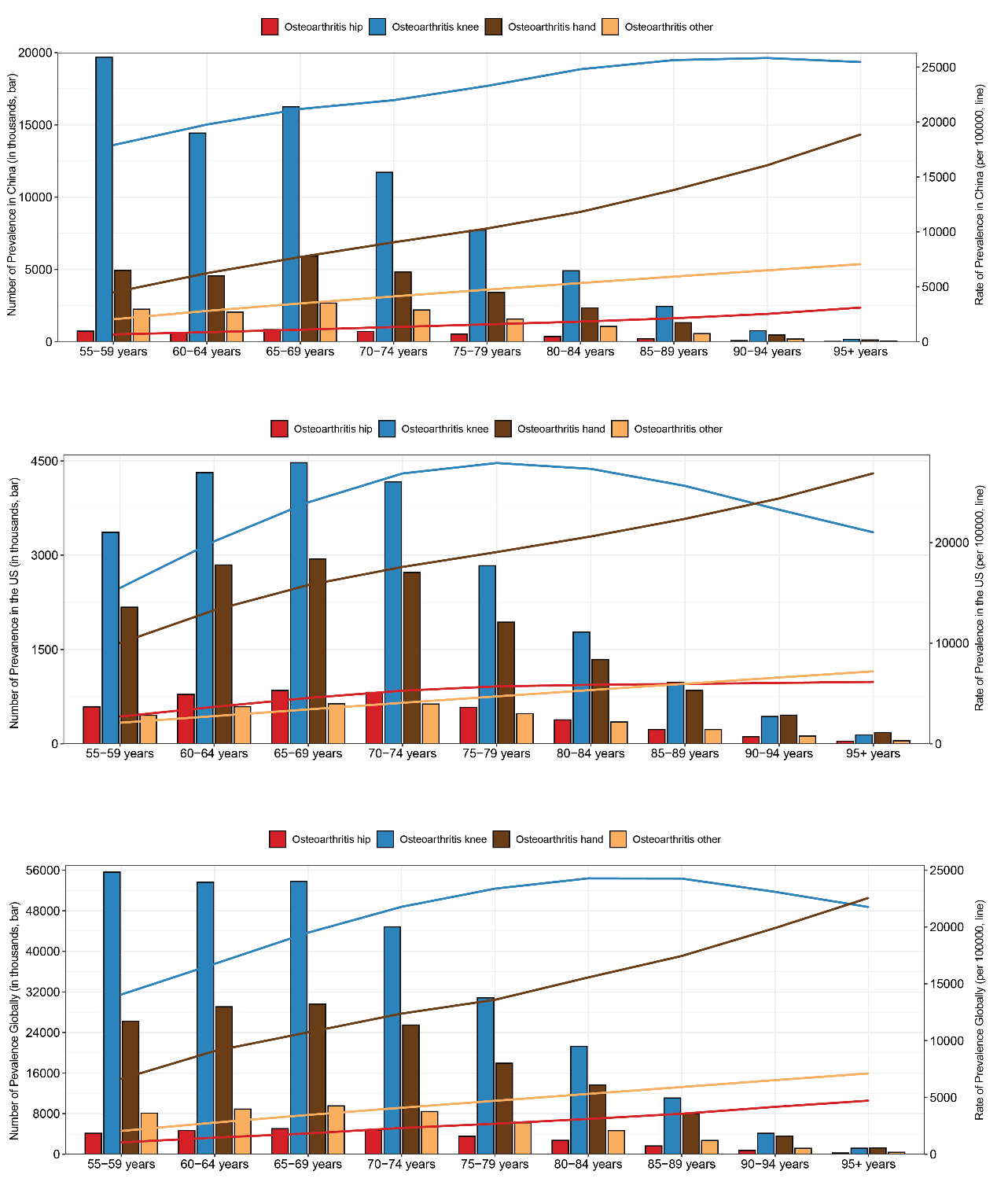
**Figure S2.** The prevalence number and rate of osteoarthritis (hip, knee, hand, and other sites) by age group in China (top), the United States (middle), and globally (bottom) in 2021.
